# The Amyloid Inhibitor CLR01 Relieves Autophagy and Ameliorates Neuropathology in a Severe Lysosomal Storage Disease

**DOI:** 10.1016/j.ymthe.2020.02.005

**Published:** 2020-02-12

**Authors:** Antonio Monaco, Veronica Maffia, Nicolina Cristina Sorrentino, Irene Sambri, Yulia Ezhova, Teresa Giuliano, Vincenzo Cacace, Edoardo Nusco, Maria De Risi, Elvira De Leonibus, Thomas Schrader, Frank-Gerrit Klärner, Gal Bitan, Alessandro Fraldi

**Affiliations:** 1 Telethon Institute of Genetics and Medicine, Via Campi Flegrei 34, 80078 Pozzuoli, Naples, Italy; 2 Institute of Cellular Biology and Biochemistry, CNR, Via Ramarini 33, 00015 Monterotondo Scalo, Rome, Italy; 3 Department of Chemistry, University of Duisburg-Essen, Universitaetsstrasse 7, 45117 Essen, Germany; 4 Department of Neurology, David Geffen School of Medicine, Brain Research Institute, University of California, Los Angeles, 635 Charles E. Young Drive South, Los Angeles, CA 90095-7334, USA; 5 Molecular Biology Institute, University of California, Los Angeles, 635 Charles E. Young Drive South, Los Angeles, CA 90095-7334, USA; 6 Department of Translational Medicine, University of Naples “Federico II,” Via Sergio Pansini 5, 80131, Naples, Italy

**Keywords:** lysosomal storage disease, autophagy, molecular tweezers, amyloid aggregation, mucopolysaccharidosis type IIIA

## Abstract

Lysosomal storage diseases (LSDs) are inherited disorders caused by lysosomal deficiencies and characterized by dysfunction of the autophagy-lysosomal pathway (ALP) often associated with neurodegeneration. No cure is currently available to treat neuropathology in LSDs. By studying a mouse model of mucopolysaccharidosis (MPS) type IIIA, one of the most common and severe forms of LSDs, we found that multiple amyloid proteins including α-synuclein, prion protein (PrP), Tau, and amyloid β progressively aggregate in the brain. The amyloid deposits mostly build up in neuronal cell bodies concomitantly with neurodegeneration. Treating MPS-IIIA mice with CLR01, a “molecular tweezer” that acts as a broad-spectrum inhibitor of amyloid protein self-assembly reduced lysosomal enlargement and re-activates autophagy flux. Restoration of the ALP was associated with reduced neuroinflammation and amelioration of memory deficits. Together, these data provide evidence that brain deposition of amyloid proteins plays a gain of neurotoxic function in a severe LSD by affecting the ALP and identify CLR01 as new potent drug candidate for MPS-IIIA and likely for other LSDs.

## Graphical Abstract

**Figure:**
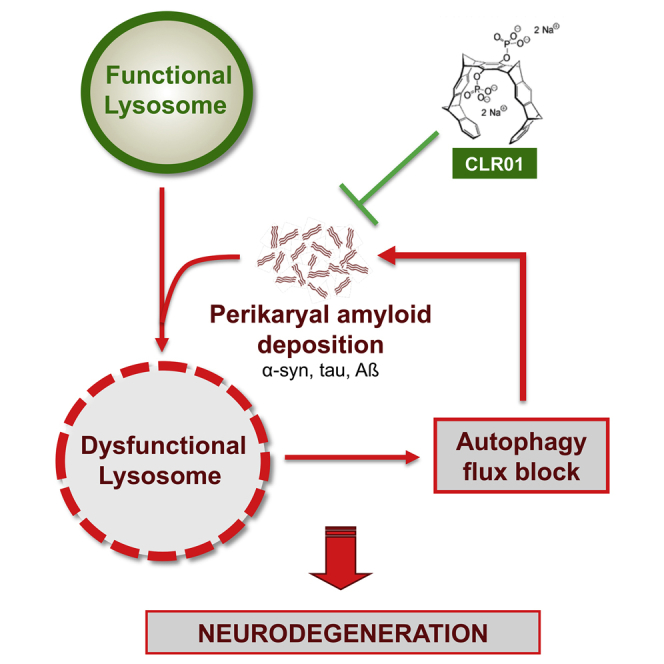

## Teaser

Fraldi and colleagues demonstrated that multiple amyloid proteins progressively aggregate in neurons of a severe lysosomal storage disease, impairing autophagy degradation and triggering neurodegeneration. They also showed that inhibiting amyloid deposition protects against neurodegeneration, thus providing evidence that amyloid aggregation is a new attractive target for the treatment of LSDs.

## Introduction

Lysosomal storage diseases (LSDs) are a group of metabolic diseases caused by inherited defects in lysosomal or non-lysosomal proteins leading to lysosomal storage and global dysfunction often associated with neurodegeneration.^1^, ^2^, ^3^ Accumulating evidence has established that in LSDs, lysosomal dysfunction is primarily characterized by an impairment of the autophagy-lysosomal pathway (ALP), one of the major intracellular clearance systems.^4^ In particular, we have shown that autophagy flux is blocked in neurodegenerative LSDs.^5^ Currently there is no cure to treat neuropathology in these diseases.

In many neurodegenerative diseases including Alzheimer’s and Parkinson’s diseases, neurotoxicity is associated with the formation of insoluble aggregates of amyloidogenic proteins, which, therefore, represent therapeutic targets for these neurological conditions.^6^ Accumulation of α-synuclein, a known amyloidogenic protein, has been shown to trigger neurotoxicity through aggregation-dependent mechanisms in Gaucher disease, a severe LSD with neurological involvement.^7^ Brain deposition of amyloidogenic proteins has been reported to occur mostly as an epiphenomenon in other neurodegenerative LSDs, including mucopolysaccharidosis (MPS) type IIIA, type IIIB, sialidosis, and Krabbe disease.^8^, ^9^, ^10^, ^11^

Recently, we have demonstrated that α-synuclein accumulates as neuronal insoluble aggregates in a mouse model of MPS-IIIA and showed that this accumulation depletes synaptic α-synuclein, contributing to neurodegeneration by a loss-of-function mechanism.^12^ Nevertheless, the neuropathogenic relevance of amyloid deposition itself and in particular its potential gain-of-toxic function in the context of LSDs remains largely unexplored.

Here, we studied amyloidogenic protein aggregation in the brain of mice with MPS-IIIA, one the most common and severe types of neurodegenerative LSDs, and found that neuronal cell bodies provide a major site for progressive deposition of multiple amyloid proteins including α-synuclein, prion protein (PrP), Tau, and amyloid-β protein (Aβ). Importantly, we demonstrated that preventing amyloid aggregation by using a broad-spectrum inhibitor of amyloid self-assembly protected against neurodegeneration in MSP-IIIA mice, thus identifying a new attractive therapeutic strategy for MPS-IIIA and likely for other LSDs.

## Results

### Multiple Amyloid Proteins Progressively Accumulate into the MPS-IIIA Mouse Brain

**Figure 1:**
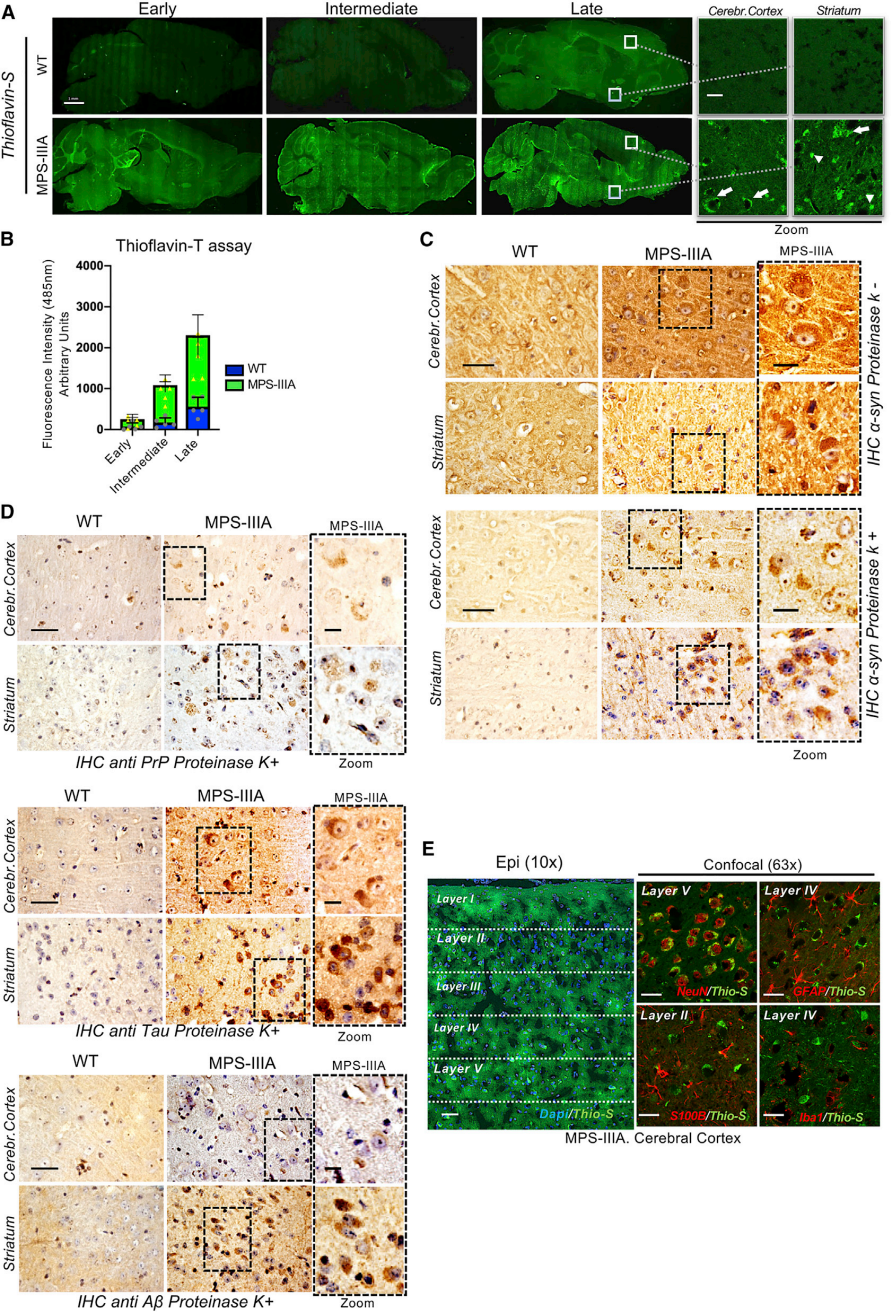
Progressive Amyloidogenic Protein Deposition in MPS-IIIA Mouse Brain (A) Sagittal brain sections of WT and MPS-IIIA mice stained with thioflavin-S (FITC filter; excitation 488 nm, emission 520 nm) at three time points: early (2–3 months), intermediate (5–6 months), and late (9–10 months). The cerebral cortex and ventral striatum areas displaying a typical staining pattern are highlighted on the right, for the late time point; the arrows indicate the perikaryal/perinuclear accumulation, whereas arrowheads indicate axonal staining. (B) Thioflavin-T fluorescence (excitation 450 nm, emission 485 nm) was quantified in WT and MPS-IIIA brain homogenates (N = 5 for each indicated time point); data were normalized to the fluorescence of brain homogenates of 1-month-old WT mice. Data are represented as means with SD. (C) α-synuclein immunohistochemistry on sagittal sections derived from the indicated brain regions of WT and MPS-IIIA mice (9 months of age) without/with proteinase-K treatment. Enlarged images of the MPS-IIIA stained sections are shown in the right panel. (D) Immunohistochemistry was performed after proteinase-K treatment on sagittal sections derived from the indicated brain regions of WT and MPS-IIIA mice (9 months of age) using specific antibodies against PrP, Tau, or Aβ. Enlarged images of MPS-IIIA brain sections are shown in the right panels. Scale bars represent 25 μm and 10 μm (zoom). (E) Immunofluorescence analysis on sagittal cortical brain sections of MPS-IIIA mice (late time point). Epi-fluorescence images (10×) show thioflavin-S co-stained with DAPI in the parietal cerebral cortex (layers I–V). Confocal images (63×) show thioflavin-S co-localization with neuronal (NeuN), astroglial (GFAP, S100β), and microglial (Iba1) markers in the indicated region. Scale bars represent 1 mm (A), 12 μm (A, zoom images), 25 μm (E, confocal images), 50 μm (E, epi-fluorescent images), 50 μm (C and D), and 5 μm (zoom images in C and D).

MPS-IIIA is caused by an inherited deficiency of sulfamidase, a lysosomal hydrolase involved in the degradation of heparan-sulfate glycosaminoglycan (GAG).^13^ Although in MPS-IIIA mice GAGs begin to accumulate into the lysosomes postnatally, ALP becomes defective only at ∼6 months of age, when severe neurological defects, including neuroinflammation, synaptic dysfunction, and cognitive deficits also appeared and then worsened progressively as the mice aged.^12^^,^^14^^,^^15^ Thioflavin-S and Congo red staining in MPS-IIIA mouse brain and thioflavin-T fluorescence assay in MPS-IIIA brain homogenates revealed a massive amyloid protein aggregation in different brain regions starting at 5–6 months of age and increasing progressively with age (Figures 1A, 1B, and S1). We have demonstrated previously that α-synuclein accumulates in the perykaria of neurons of MPS-IIIA mice.^12^ To test whether α-synuclein was a component of the observed amyloid deposits, we performed immunohistochemistry (IHC) analysis with proteinase-K digestion. This analysis showed that α-synuclein inclusions found in MPS-IIIA mouse brain were resistant to the digestion, a key feature of amyloid deposits (Figures 1C and S2A). We tested next whether other proteins also were present in the amyloid deposits by using antibodies against different known aggregation-prone amyloidogenic proteins, including Tau, PrP, Aβ, huntingtin, and TAR DNA-binding protein (TDP-43).^16^ A strong proteinase K-resistant immunoreactivity was found for Tau, PrP, and Aβ in several brain regions of the MPS-IIIA mice (Figures 1D, S2B, and S2C). Confocal-microscopy analysis showed that brain inclusions of these proteins were, for the most part, also thioflavin-S-positive, supporting their amyloid nature (Figure S3). Co-labeling of thioflavin-S with specific markers of glial and neuronal cells showed that a major part of the thioflavin-S signal localized in the perikaryal region of neurons (Figures 1E and S4). Thioflavin-S fluorescence and amyloid protein immunoreactivity signals were also analyzed in terms of subcellular localization and shape. Although a fraction of the signal could be detected as characteristic axonal spheroids or filamentous deposits, the majority was found as cell body inclusions (Table S1). Thus, multiple amyloid proteins progressively deposit in the brain of MPS-IIIA mice mainly localizing in neuronal cell bodies.

### The Molecular Tweezer CLR01 Efficiently Inhibits Amyloid Protein Aggregation in the MPS-IIIA Mouse Brain

**Figure 2:**
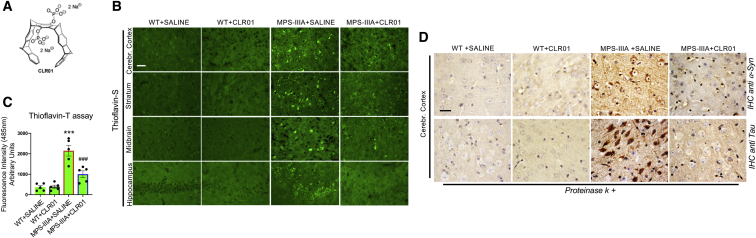
CLR01 Treatment Inhibits Amyloid Deposition in the Brain of MPS-IIIA Mice (A) Chemical structure of CLR01 showing the aromatic backbone and two phosphate groups, which confer the specificity for binding to positively charged amino acid side chains. (B) CLR01 was injected daily at 1 mg/Kg subcutaneously to 4.5-month-old WT and MPS-IIIA mice over a period of 4.5 months. As a control, WT and MPS-IIIA mice were treated with saline. Thioflavin-S staining was performed at the end of treatment (9 months of age) on sagittal sections derived from the indicated brain regions of the four experimental groups of mice (WT saline-injected, WT CLR01-injected, MPS-IIIA-saline injected, and MPS-IIIA CLR01-injected) showed a reduction of amyloid deposition in the brain of CLR01 treated MPS-IIIA mice. (C) Thioflavin-T fluorescence (excitation 450 nm, emission 485 nm) was quantified in the brain homogenates derived from the four experimental groups of mice (N = 5 for each indicated time point); data were normalized to the fluorescence of brain homogenates of 1-month-old WT mice. ***p < 0.001 versus WT; ###p < 0.001 versus MPS-IIIA + saline, one-way ANOVA with Bonferroni’s multiple comparison test. Data are represented as means ± SEM. (D) Immunohistochemistry with proteinase-K treatment was performed at 9 months of age on sagittal cortical sections of mice belonging to the four experimental groups using antibodies against specific amyloid proteins (α-synuclein and tau are shown). A strong reduction in proteinase K-resistant aggregates was found in MPS-IIIA brain treated with CLR01 compared to MPS-IIIA brain treated with saline. Scale bars represent 50 μm (B) and 12.5 μm (D).

To address whether amyloid aggregation in MPS-IIIA mice was relevant for neuropathogenesis, we treated mice with a broad-spectrum inhibitor of amyloid protein oligomerization and aggregation, the “molecular tweezer” CLR01 (Figure 2A) and studied its impact on neuropathology. Molecular tweezers act by a process-specific mechanism binding to lysine residues and disrupting molecular interactions that are important for the abnormal self-assembly of multiple amyloidogenic proteins.^17^^,^^18^ The lead molecular tweezer CLR01 was selected out ∼10 first-generation molecular tweezers derivatives, based on its low toxicity and high efficacy inhibiting Aβ-induced toxicity in cell culture. It was also evaluated for its pharmacokinetic characteristics and safety in mice using both acute and chronic administration.^19^ CLR01 was shown to be very effective in protecting against neurodegeneration by inhibiting protein aggregation in mouse models of synucleinopathies, Alzheimer’s disease (AD), and transthyretin amyloidosis.^20^, ^21^, ^22^, ^23^ Importantly, systemic administration of CLR01 is safe in mice and allows for efficient brain targeting due to the capability of CLR01 to cross the blood-brain barrier.^19^ We treated the MPS-IIIA mice starting at 4.5 months of age, before massive amyloid aggregation takes place, with daily sub-cutaneous injection of CLR01 over a period of 4.5 months. At the end of treatment, 9-month-old MPS-IIIA mice showed a strong reduction in thioflavin-S and thioflavin-T reactivity (Figures 2B and 2C) and, correspondingly, a striking decline in the build-up of specific components of amyloid deposits (Figure 2D).

### CLR01 Treatment Relieves Autophagy-Lysosomal Dysfunction in the MPS-IIIA Mouse Brain

**Figure 3:**
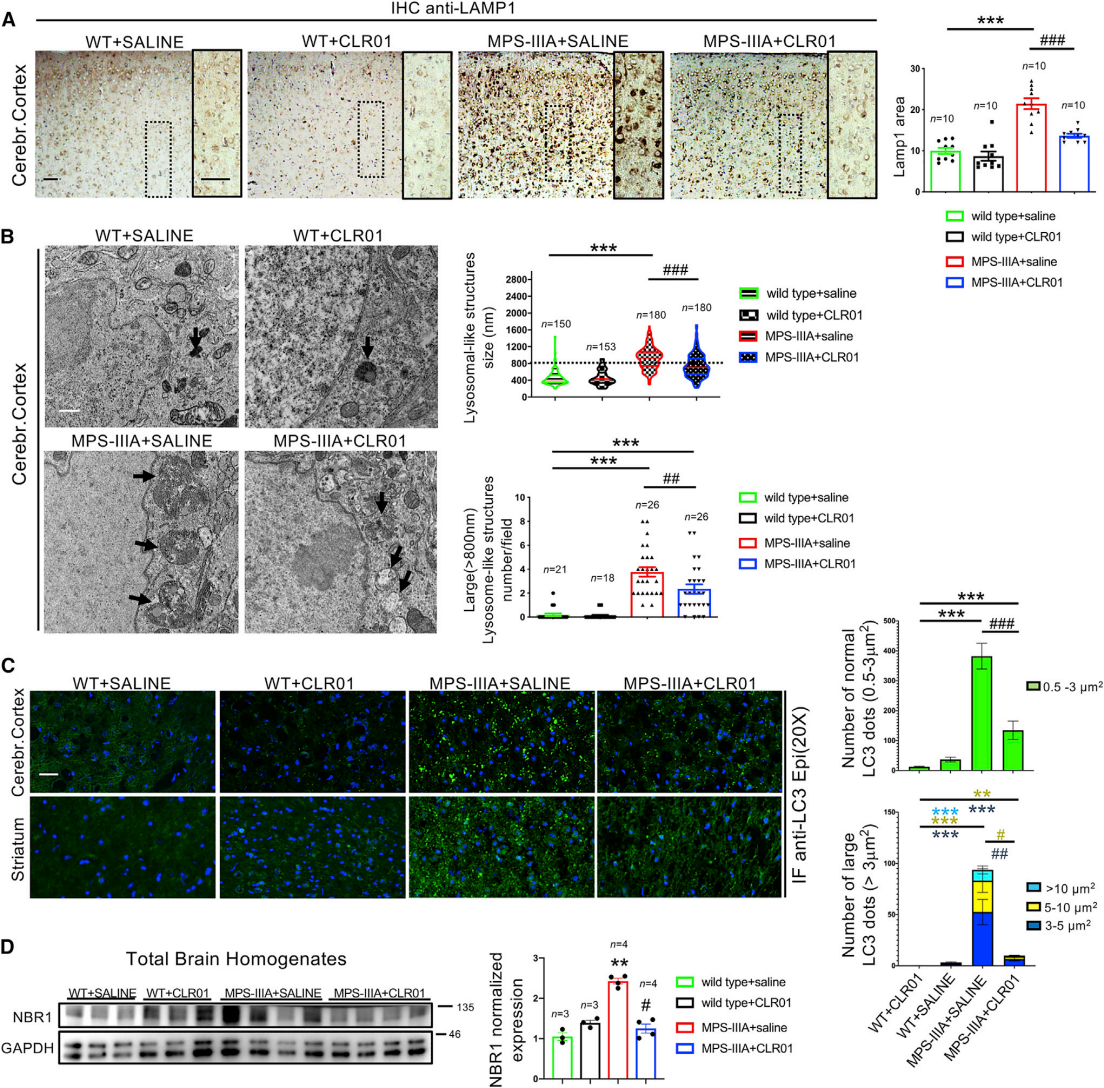
CLR01 Treatment Reduces Lysosomal Enlargement and Restores Autophagy Flux in the Brain of MPS-IIIA Mice (A) Immunohistochemistry using an anti-Lamp1 antibody was performed on sagittal brain sections derived from the four experimental groups of mice at 9 months of age. Images from cerebral cortex are shown. Lamp1 immunoreactivity was quantified in 10 different fields from cortical regions for each group. Three mice were analyzed for each experimental group. ***p < 0.001 versus WT; ###p < 0.001 versus MPS-IIIA + saline, one-way ANOVA with Bonferroni’s multiple comparison test. Data are represented as means ± SEM. (B) Transmission electron microscopy (TEM) analysis was used to quantify the number and size of lysosomal-like structures (black arrows) in the four experimental groups of mice at 9 months of age. For size measurement, at least 150 lysosomes from the cortical regions for each group were analyzed. For number measurement, ~20 fields from the cortical regions for each group were analyzed. Three mice were analyzed per group. Representative TEM images are shown on the left panel. ***p < 0.001 versus WT; ##p < 0.01, ###p < 0.001 versus MPS-IIIA + saline, respectively, one-way ANOVA with Bonferroni’s multiple comparison test. Data are represented as means ± SEM. (C) Immunofluorescence using an anti-LC3 antibody was performed on indicated sagittal brain sections from the four experimental groups of mice at 9 months of age. Large LC3-positive puncta (>3 μm^2^) were considered as area containing several accumulating AVs. LC3 puncta ranging from 0.5 to 3 μm^2^ were considered as area containing normal sized AVs. The number of LC3-positive puncta was quantified in 15 different fields from the cortical regions for each group. Three mice were analyzed per group. ***p < 0.001 versus WT; ###p < 0.001 versus MPS-IIIA + saline; ##p < 0.01 versus MPS-IIIA; #p < 0.05 versus MPS-IIIA, one-way ANOVA with Bonferroni’s multiple comparison test. Data are represented as means ± SEM. (D) Western-blot analysis of the autophagy markers NBR1 was performed in the brain homogenates derived from the four experimental mouse groups at 9 months of age. NBR1 signal was quantified and normalized to GAPDH signal. **p < 0.01 versus WT; #p < 0.05 versus MPS-IIIA, one-way ANOVA with Bonferroni’s multiple comparison test. Data are represented as means ± SEM. Scale bars represent 25 μm (A and C) and 0.4 μm (B).

Lysosomal enlargement and autophagy dysfunction are associated to neurodegeneration in several LSDs, including the MPS-IIIA.^2^^,^^4^ Consistent with the large expansion of the lysosomal compartment, a pathological hallmark of LSDs, anti-LAMP1 IHC showed an increased signal in MPS-IIIA mouse brain compared to age-matched wild-type (WT) mice (Figure 3A). Electron microscopy (EM) analysis revealed that this increased LAMP1 immunoreactivity reflected enlargement of the average lysosome size associated with an increase in the number of abnormal-sized lysosomes (>800 nm) at the expense of normal-sized lysosomes (∼400 nm) (Figure 3B). CLR01 treatment markedly reduced the enlargement of the lysosomal compartment in the MPS-IIIA mouse brain (both the average size and the number of abnormal-sized lysosomes were reduced) but did not modify it in WT mice (Figures 3A and 3B).

We then analyzed the autophagy flux in the MPS-IIIA mouse brain following CLR01 treatment. In healthy neurons, autophagosome vesicles (AVs) are continuously generated in the axons and efficiently cleared upon fusion with lysosomes in the cell body and, therefore, are rarely found in neurites or cell bodies unless lysosome-dependent AV clearance is disrupted.^24^, ^25^, ^26^ Immunofluorescence analysis of the AV marker LC3 showed a striking increase in the overall number of LC3-positive puncta in MPS-IIIA brain compared to WT mouse brain (Figure 3C). Interestingly, the majority of LC3-puntca were not within the soma of cells (Figure 3C). This result was consistent with a block of the autophagic flux, as shown in previous reports.^12^^,^^27^ CLR01 treatment significantly decreased the overall number of LC3-positive puncta in the MPS-IIIA brain, indicating that lysosomal-mediated clearance of autophagosomes was efficiently restored by inhibiting amyloid deposition (Figure 3C). Importantly, no significant change in LC3 staining was observed in CLR01-treated WT mice (Figure 3C). We also monitored the levels of NBR1, an autophagy cargo receptor.^28^ Western-blot anti-NBR1 showed an accumulation of NBR1 protein in the brain of MPS-IIIA mice that was significantly reduced upon CLR01 treatment (Figure 3D), thus further supporting the conclusion that inhibition of amyloid deposition by CLR01 reactivated the autophagic flux in MPS-IIIA brain.

### CLR01 Treatment Ameliorates Neurodegenerative Signs in MPS-IIIA Mice

**Figure 4:**
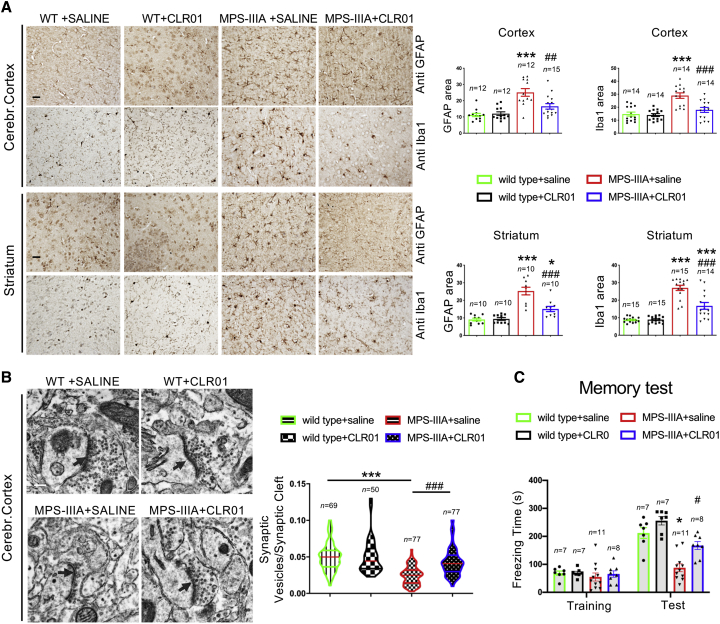
CLR01 Treatment Ameliorates Neuropathological Signs in MPS-IIIA Mice (A) Immunohistochemistry with anti-GFAP and anti-Iba-1 antibodies was performed on sagittal both cortical and striatal brain sections derived from mice belonging to the four experimental groups at 9 months of age. Images show a reduction of both inflammatory markers in MPS-IIIA mice upon CLR01 treatment. GFAP and Iba1 immunoreactivity were quantified in 10–15 different fields. Three mice for each experimental group were analyzed. *p < 0.05 versus WT + saline; ***p < 0.001 versus WT + saline; #p < 0.05, ##p < 0.01, ###p < 0.001 versus MPS-IIIA + saline; one-way ANOVA with Bonferroni’s multiple comparison test. Data are represented as means ± SEM. (B) TEM analysis of synaptic-vesicle number was performed in the cerebral cortex of mice belonging to the four experimental groups at 9 months of age. The graphic representation shows the synaptic vesicles/synaptic cleft length ratio for at least 50 synapses derived from 3 mice for each experimental group. The arrows indicate the synaptic cleft. ***p < 0.001 versus WT + saline; ###p < 0.001 versus MPS-IIIA + saline, one-way ANOVA analysis with Bonferroni’s multiple comparison test. (C) Memory function was tested in mice at 9 months of age using the contextual fear-conditioning task. Three different cohorts of injected animals were used. Graphical representation of contextual fear-conditioning results (freezing time during training and test) show that memory deficits in control MPS-IIIA mice (reduced freezing time) were rescued by CLR01 treatment. 7–11 male mice in each group were tested. Repeated-measures ANOVA: Genotype F_1,29_ = 23.046; p < 0.0001; Treatment F_1,29_ = 7.862; p = 0.0089; Freezing F_1,29_ = 241.926; p < 0.0001; Freezing × Genotype F_1,29_ = 42.580; p < 0.0001; Freezing × Treatment F_1,29_ = 14.086; p = 0.0008. *p < 0.05 versus WT; #p < 0.05 versus MPS-IIIA, Duncan post hoc test. WT saline (n = 7), WT + CLR01 (n = 7), MPS-IIIA saline (n = 11), MPS-IIIA + CLR01 (n = 8). Data are represented as means ± SEM. Scale bars represent 25 μm (A) and 0.2 μm (B).

Next, we tested whether CLR01-mediated restoration of ALP in MPS-IIIA brain was associated with a neuroprotective effect. Neuro-inflammation is one of the most prominent signs of neuropathology in MPS-IIIA.^29^^,^^30^ We found that CLR01 treatment strongly reduced the neuro-inflammation in the MPS-IIIA mice while it had no effect on WT mice (Figure 4A). EM analysis of cortical synapses showed that CLR01 treatment also rescued loss of synaptic vesicles (Figure 4B), another pathological feature that has been reported in MPS-IIIA mouse brain.^12^^,^^29^ Recent data demonstrated that MPS-IIIA mice develop a strong age-dependent memory impairment in the contextual fear-conditioning test.^31^ Indeed, untreated 9-month-old MPS-IIIA mice showed a severe memory deficit compared to WT mice, which, remarkably, was fully rescued by CLR01 treatment (Figure 4C). Taken together, our data showed that CLR01-mediated inhibition of amyloid formation in MPS-IIIA by CRL01 mice restores ALP function and reduces neuropathological signs significantly, thus indicating that amyloid deposition contributes to the neurodegenerative processes in a relevant model of neurodegenerative LSDs. To support these findings, we also tested whether CLR01 also could improve neuropathology when administered at a late stage of disease progression, when amyloid deposition had already occurred. To this aim, we treated MPS-IIIA at 7 months of age continuously until 11 months of age. This treatment regimen did not affect the buildup of amyloid inclusions and did not improve significantly neither the ALP functionality nor neurodegenerative signs (Figure S5). Therefore, CLR01 was effective in preventing amyloid aggregation but not in destroying preexisting aggregates. Moreover, these data further support the functional relationship between amyloid accumulation, ALP dysfunction, and neurodegenerative processes in MPS-IIIA.

## Discussion

Targeting secondary pathogenic processes may represent a valuable option for the treatment of LSDs that is alternative to the correction of the primary genetic defect.^32^ Finding effective druggable therapeutic targets may also open the possibility of improving existing therapeutic protocols and, in principle, may be applied to several LSDs.^33^

Our data demonstrate that multiple amyloid proteins massively accumulate mainly in neuronal cell bodies of a mouse model of MPS-IIIA, a severe neurodegenerative LSD, and that inhibiting amyloid deposition by CLR01 relieves ALP dysfunction and ameliorates neurological signs in MPS-IIIA mice. Importantly, CLR01 effect on autophagy must have been mediated by the inhibition of amyloid deposition because no appreciable change in the lysosomal-autophagic axis was observed in WT mice upon CLR01 treatment, thus ruling out a direct effect of CLR01 on ALP. Therefore, our data pointed out on a neurotoxic effect of amyloid deposition in MPS-IIIA that is associated to the impairment of ALP, thus identifying amyloid aggregation as a new therapeutic target for MPS-IIIA. How amyloid protein deposition affects ALP remains to be elucidated. Mechanisms underlying such effects may involve the negative impact of amyloids on lysosomal trafficking and signaling pathways, which have been shown to be critical for autophagy.^26^^,^^34^ The finding that LC3 puncta accumulate out of cell bodies (likely in neurites) suggests a mechanism by which amyloid deposition impairs autophagosome maturation. A point that also needs to be clarified is what initiates amyloid protein deposition. One possibility is that primary lysosomal storage induces amyloid protein deposition. Supporting this hypothesis, it has been reported that GAGs provide a scaffold promoting amyloid aggregation.^35^^,^^36^ Moreover, the autophagy block may, in turns, boost amyloid build-up, thus generating a vicious cycle that can be interrupted by CLR01 treatment.

In conclusion, our data showed that the brain deposition of multiple amyloidogenic proteins plays a key role in the onset of neuropathology in a severe form of neurodegenerative LSDs and that the amyloid self-assembly inhibitor CLR01 can efficiently protect against neurological lesions. Importantly, our data indicate that the neuroprotective effect of CLR01 is associated with the restoration of ALP. These findings provide new insights into the processes triggering neurodegeneration in LSDs and identify amyloid deposition counteracting as a new attractive therapeutic strategy for MPS-IIIA and likely for other LSDs.

## Materials and Methods

### Animals

MPS-IIIA mice (homozygous mutant *Sgsh*^−/−^)^15^, together with respective control littermate WT (+/+), were utilized. All mice were C57BL/6 congenic. Animal studies were conducted in accordance with the guidelines of the Animal Care and Use Committee of TIGEM in Naples and authorized by the Italian Ministry of Health.

### CLR01 Injection

CLR01 was synthesized in the lab of Thomas Schrader and Frank-Gerrit Klärner, both at the University of Duisburg-Essen (UDE) in Germany.^37^ The corresponding composition of matter and methods of use intellectual property (IP) are held jointly by UCLA (Gal Bitan) and UDE (Thomas Schrader and Frank-Gerrit Klärner) in International Patent No. PCT/US2010/026419, USA patent No. 8,791,092, European patent No. EP2403859 A2 (Massachusetts Institute of Technology is a minor partner), and International Patent Application No. PCT/US2019/039943 (Ulm University, Germany is also a partner). The method of use applied to lysosomal storage diseases that was discovered by Alessandro Fraldi at the Telethon Institute of Genetics and Medicine (TIGEM), Italy, is protected by International Patent Application No. PCT/US2019/029222, which is held jointly by TIGEM and UCLA. CLR01 powder was dissolved in phosphate-buffered saline (PBS pH 7.4) at concentration of 0.7 mg/mL. Each day the MPS-IIIA and WT mice were weighed and a volume of the CLR01 solution corresponding to 1 mg/kg/day (approximately 50–60 μL of solution per mouse) was subcutaneously injected. Control mice, both MPS-IIIA and WT, were treated similarly with PBS only.

### Brain Collection

After euthanizing, mouse brains were collected from each experimental group and perfused with PBS to clear blood from the tissue. The brains were divided into the two hemispheres: one was frozen in dry ice and used for biochemical analysis and the other was fixed in 4% (w/v) paraformaldehyde in PBS and embedded in optimal cutting temperature compound (OCT compound) or paraffin.

### Brain Homogenization and Protein Extraction

Brain hemispheres were ruptured in 6 volumes of milli-Q water containing Protease Inhibitor Cocktail Set I (Calbiochem) by using the TissueLyser LT (QIAGEN). Lysates were subjected to five freeze-thaw cycles. Water homogenates were used for thioflavin T assays.

Brain homogenates were also used for proteins extraction performed by radioimmunoprecipitation assay (RIPA) buffer containing 1% Triton X-100 and Protease Inhibitor Cocktail Set I (Calbiochem). Samples were subjected to a centrifugation at 10,000 rpm for 10 min, and the supernatants were collected as protein-enriched solutions. Protein samples were loaded onto a 12% SDS-PAGE in order to analyze NBR1 expression by western blotting.

### Thioflavin T Assay

The brain homogenates were sonicated and quantified for protein content by Bradford assay. 50 μg of homogenate were incubated for 5 min at room temperature with thioflavin T (ab120751, Abcam) prepared at 20 μM in water. Fluorescence emission at 485 nm (excitation at 450 nm) then was measured at 37°C. The fluorescence values were normalized by subtracting the fluorescence coming from brain homogenates derived from 1-month-old WT mice.

### Antibodies

Polyclonal rabbit antibody anti-α-synuclein (#128102, Synaptic System, Goettingen, Germany, IHC, IF: 1:300), polyclonal goat antibody anti-PrP (ab6664, Abcam, Cambridge, UK, IHC, IF: 1:400), monoclonal mouse antibody anti-Tau (Tau 46) (#4019, Cell Signaling Technology, Danvers, USA, IHC, IF: 1:400), monoclonal rabbit antibody anti-Aβ 1-42 conformation specific (ab201060, Abcam, IHC, IF: 1:200), polyclonal rabbit antibody anti-TDP-43 (#10782-2AP, Proteintech, Chicago, USA, IHC: 1:200), monoclonal rabbit antibody anti-huntingtin (ab109115, Abcam, IHC: 1:100), polyclonal rabbit antibody anti-glial fibrillary acidic protein (GFAP) (Z0334, DAKO Agilent, Santa Clara, USA, IF, IHC: 1:400), polyclonal rabbit antibody anti-IBA 1 (019-19741, Wako, USA, IHC, IF: 1:200), polyclonal rabbit antibody anti-LC3 (PM036, MBL, Woburn, USA, IF: 1:1,000), monoclonal rat antibody anti-Lamp1 (SC-19992, Santa Cruz, Dallas, USA, IF: 1:50), polyclonal rabbit antibody anti-Lamp1 (ab24170, Abcam, IHC: 1:200), polyclonal rabbit antibody anti-S-100β (ab52642, abcam, IF: 1:200), monoclonal mouse antibody anti-NBR1 (H00004077-M01, Abnova, Taiwan, WB: 1:1,000); monoclonal mouse antibody anti-NeuN (MAB377, Millipore, IF: 1:400), and monoclonal mouse antibody anti-Olig-2 (MABN50, Millipore, IF: 1:200). Alexa Fluor secondary antibodies used in immunofluorescence experiments were purchased from Molecular Probe (Invitrogen).

### Proteinase K Treatment

We used a standard protocol as in Roberts et al.^38^ Briefly, after rehydration, 6-μm thick paraffin sections were treated with 3% H_2_O_2_ for 1 h to quench the endogenous peroxidases. Proteinase K (Euroclone) was added onto the paraffin sections at a concentration of 50 μg/mL for 2 min at 37°C. If indicated, unmasking was eventually performed by incubating sections for 10 min in a citrate buffer, pH 6, in a microwave oven. After blocking, sections were incubated with the specific antibodies.

### Immunofluorescence and Immunohistochemistry

Medial sagittal 10 μm sections of frozen brain tissue were cut using a cryostat, permeabilized in PBS containing 0.05% Saponin, and 10% fetal bovine serum), and incubated with the appropriate primary antibody overnight at +4°C followed by 1 h at room temperature with the secondary antibody. Stained sections were mounted with Vectashield (Vector Laboratories, CA, USA). Sections were analyzed using a Leica upright DM5500 microscope or a confocal Zeiss LSM 700 microscope using either 63× or 40× objectives.

The percentage of colocalization was evaluated on confocal images by using the tool “Colocalization Test” of the ImageJ software.

Analysis of LC3 dots was performed by using the tool “analyze particles” of the ImageJ software. Counting of dots was performed using a circularity of 0.5–1. Because AV size normally ranges from 0.2 to ∼2–3 μm,^2^^,^^39^ dots smaller than 0.2 μm^2^ were not considered in the analysis. The LC3 dots were divided in two different populations based on their size; dots ranging from 0.2 to 3 μm^2^ were considered normal and dots greater than 3 μm^2^ were considered large. Moreover, the large LC3 dots were further divided in three different classes from 3 μm^2^ to 5 μm^2^, 5 μm^2^ to 10 μm^2^, and >10 μm^2^.

For paraffin embedding, mouse brains were fixed in 4% paraformaldehyde (PFA) overnight at 4°C, then dehydrated in a graded series of ethanol, cleared with xylene, and infiltrated with paraffin. Paraffin-embedded blocks were cut on a microtome into 6-μm sagittal sections. All immunohistochemistry staining was performed using the Vectastain Elite ABC-HRP Kit (PK6200 Vector Laboratories). Visualization was performed using 3,3-diaminobenzidine tetrahydrochloride (DAB Vector Laboratories). Bright-field images were observed using an upright Leica DM5500 microscope. Quantitation of the stained area in immunohistochemistry experiments was performed by using the tool “measure” of ImageJ after the conversion of images to 8-bit format and the adjustment of the threshold.

### Thioflavin-S Staining

Cryo-sections (10 μm) were incubated in 0.01% (w/v) thioflavin-S (T1892 SIGMA) for 10 min. Slides were washed in PBS 3 times and mounted using Vectashield mounting medium with 4′,6-diamidino-2-phenylindole (DAPI; Vector Laboratories, CA, USA). Positive staining was observed using an upright Leica DM5500 microscope with the fluorescein isothiocyanate (FITC) filter. For the scan of the entire sections, the images were acquired by Scan-Scope slide scanner (Leica scn400).

### Congo Red Staining

Paraffin sagittal brain sections (6 μm) were deparaffinized, hydrated, and stained with Mayer’s hematoxylin solution for 10 min. After a rinse in tap water for 5 min, the sections were placed in alkaline sodium chloride solution (Sigma-Aldrich, HT60) for 20 min and then stained for 20 min in a Congo red solution (Sigma-Aldrich, HT60). The sections were rinsed 3 times in absolute ethanol, cleared in xylene, and mounted. The images were acquired using an upright Leica DM5500 microscope.

### Electron Microscopy

Cortex regions were fixed in 1% glutaraldehyde and 4% PFA in 200 mM HEPES, pH 7.3, for 10 min at 37°C, post-fixed in 1% osmium tetroxide, dehydrated, and embedded in resin. Ultra-thin sections were cut on a LEICA EM UC7 ultramicrotome and the morphology of cellular and subcellular structures was analyzed by the TIGEM EM core. Lysosome size and number were evaluated using the software iTEM (Olympus). “Lysosomes” were identified by morphometric analysis as a wide spectrum of lysosome-like structures that includes bona fide lysosomes, autolysosomes, multi-vesicular bodies, and vacuoles with stored undigested material.

### Contextual Fear Conditioning Test

The contextual fear conditioning task allows to evaluate the mouse ability to learn and remember a Pavlovian association between a mild food-shock and a specific context. The test was carried out in a behavioral testing room maintained under constant light, temperature, and humidity. The mice were tested during daylight hours (between 9 am and 6 pm). Before testing, animals were habituated to the testing room for at least 30 min.

Each mouse was trained in a conditioning chamber (30 cm × 24 cm × 21 cm; Ugo Basile) that had a removable grid floor and waste pan. The grid floor contained 36 stainless steel rods (3-mm diameter) spaced 8 mm center to center. When placed in the chamber, the grid floor contacted a circuit board through which scrambled shock was delivered. The shock intensity was 0.5 mA with a duration of 2 s and it was presented three times and was associated with a context. 24 h after training, mice were tested without foot-shock but with the same context. Freezing behavior was defined as a complete lack of movement, except for respiration, and scored using a video-tracking system (ANY-MAZE, Stoelting, USA).

### Statistical Analysis

Data of behavioral tests were expressed as mean ± SEM. Two-way ANOVA for repeated-measures with the factor group as independent factor and trials or test phase as repeated-measures was performed to assess significance among multiple experimental groups and at different time points, followed by Duncan post hoc test when appropriate. A p < 0.05 was considered as statistically significant.

For all the other experiments, the data were expressed as mean ± SEM. The significance of difference among the experimental groups were calculated by one-way ANOVA followed by Bonferroni post hoc test. A p < 0.05 was considered as statistically significant.

## Author Contributions

A.M. performed experimental procedures, analyzed data, and contributed to the writing of the manuscript. V.M contributed to all experimental procedures. N.C.S contributed to IHC, EM experiments, and injections in mice. Y.E. contributed to setting thioflavin staining. I.S. and T.G. generated preliminary data on CLR01 short-term effect *in vivo.* M.D.R. performed behavioral testing and analyzed behavioral data. T.S. and F.-G.K. invented and synthesized the molecular tweezers used herein. E.N. helped with mice experiments. E.D.L. designed and supervised behavioral experiments and contributed to writing the manuscript. G.B. contributed to the design of the study and edited the manuscript. A.F. designed and supervised research and wrote the manuscript.

## Conflicts of Interest

The authors declare no competing interests.

## Supplemental Information

Document S1. Figures S1–S5 and Table S1

Document S2. Article plus Supplemental Information
