# The Amyloid Inhibitor CLR01 Relieves Autophagy and Ameliorates Neuropathology in a Severe Lysosomal Storage Disease

**DOI:** 10.1016/j.ymthe.2022.10.001

**Published:** 2022-10-18

**Authors:** Antonio Monaco, Veronica Maffia, Nicolina Cristina Sorrentino, Irene Sambri, Yulia Ezhova, Teresa Giuliano, Vincenzo Cacace, Edoardo Nusco, Maria De Risi, Elvira De Leonibus, Thomas Schrader, Frank-Gerrit Klärner, Gal Bitan, Alessandro Fraldi

## Main text

(Molecular Therapy *28*, 1167–1176; April 2020)

In the originally published version of this article, an error was made during the assembly of Figure 4A: the cropped image used as representative of "striatum anti-GFAP WT + saline" was inadvertently taken from the folder containing the cropped images of "striatum anti-GFAP WT + CLR01" fields, instead of from the correct folder (cropped images of “striatum anti-GFAP WT + saline” fields). Therefore, two partial overlapping cropped images of the same field (striatum anti-GFAP WT + CLR01) were used in the panel.

The correct version of Figure 4A is included here. The authors apologize for the error.

**Figure:**
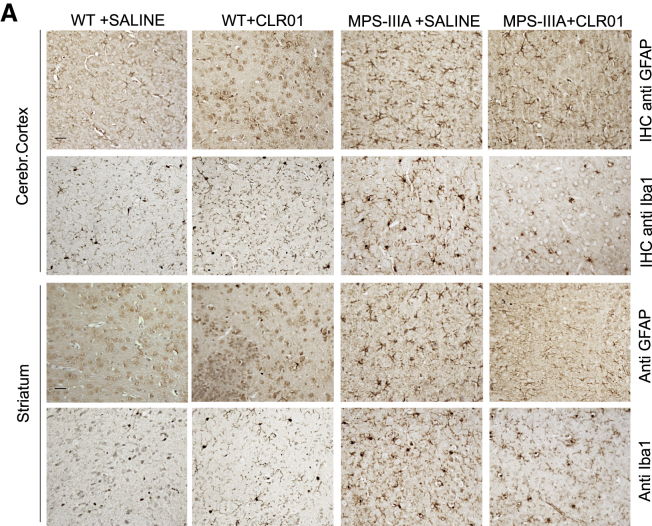
Figure 4A. CLR01 Treatment Ameliorates Neuropathological Signs in MPS-IIIA Mice (corrected)

