# Supplementary material for: The Amyloid Inhibitor CLR01 Relieves Autophagy and Ameliorates Neuropathology in a Severe Lysosomal Storage Disease
Source: Mol Ther. 2020 Feb 12;28(4):1167–76. doi: 10.1016/j.ymthe.2020.02.005 (PMC7132627; doi:10.1016/j.ymthe.2020.02.005)
Supplement: Document S1. Figures S1–S5 and Table S1 [file mmc1.pdf]

YMTHE, Volume *28*

Supplemental Information

The Amyloid Inhibitor CLR01 Relieves Autophagy and Ameliorates Neuropathology in a Severe Lysosomal Storage Disease

Antonio Monaco, Veronica Maffia, Nicolina Cristina Sorrentino, Irene Sambri, Yulia Ezhova, Teresa Giuliano, Vincenzo Cacace, Edoardo Nusco, Maria De Risi, Elvira De Leonibus, Thomas Schrader, Frank-Gerrit Klärner, Gal Bitan, and Alessandro Fraldi

The amyloid self-assembly inhibitor CLR01 relieves autophagy and ameliorates neuropathology in a severe lysosomal storage disease

Supplemental material

- Supplemental figures
- Supplemental figure legends
- Supplemental Tables

Supplementary Figure S1

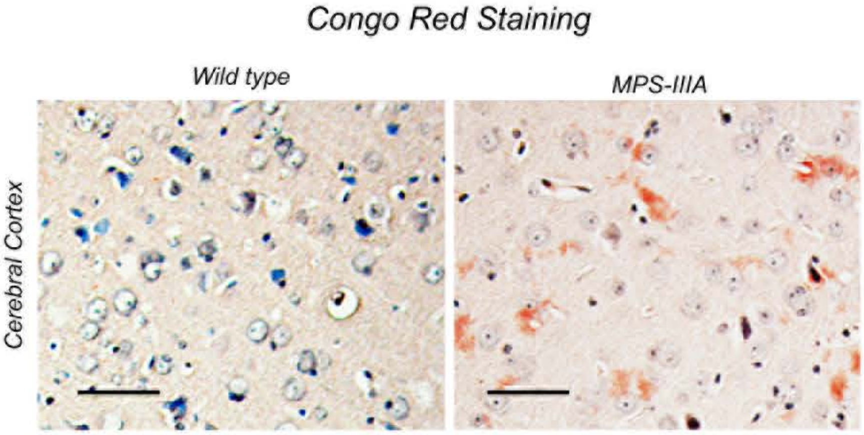

Supplementary Figure S2

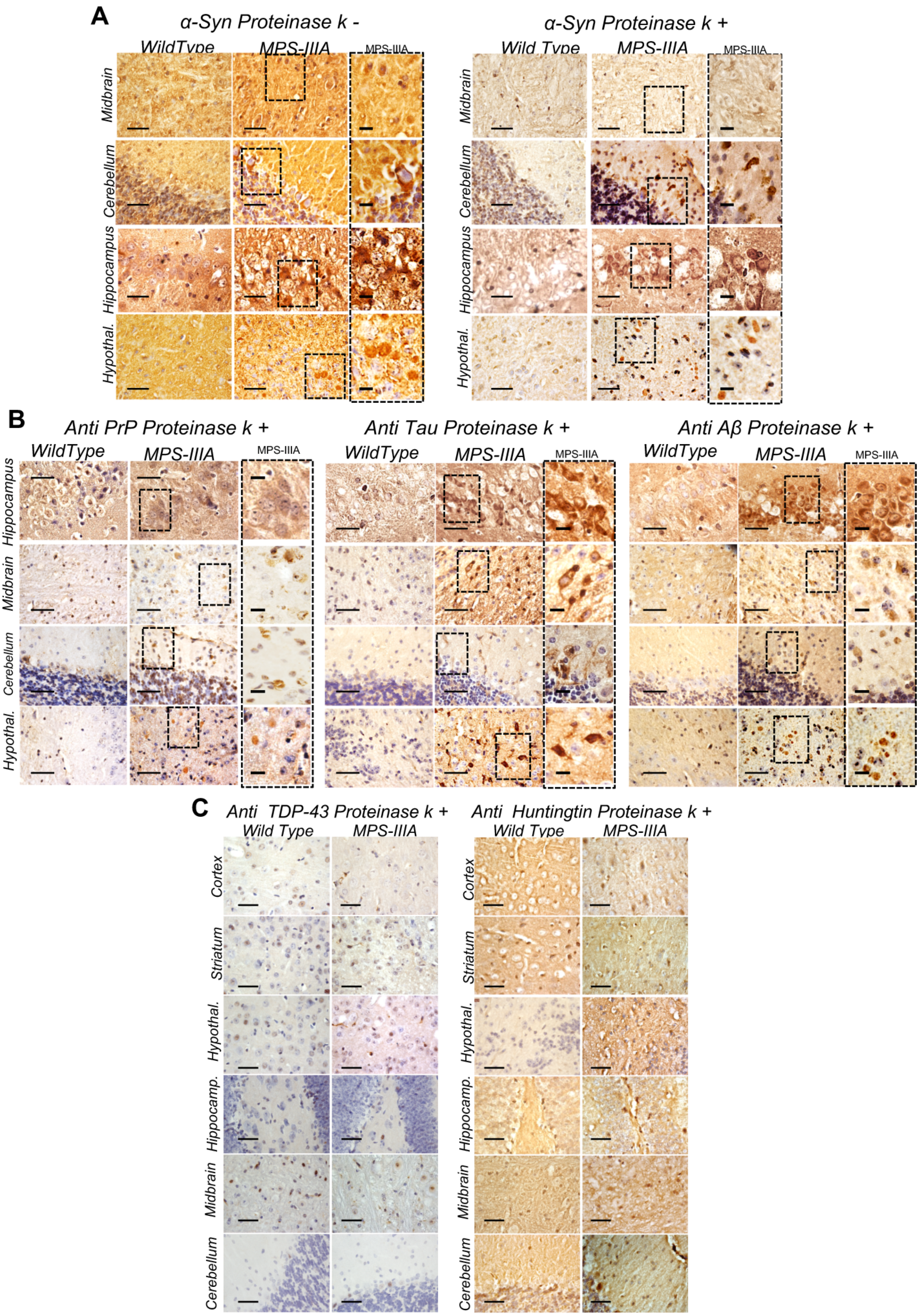

Supplementary Figure S3

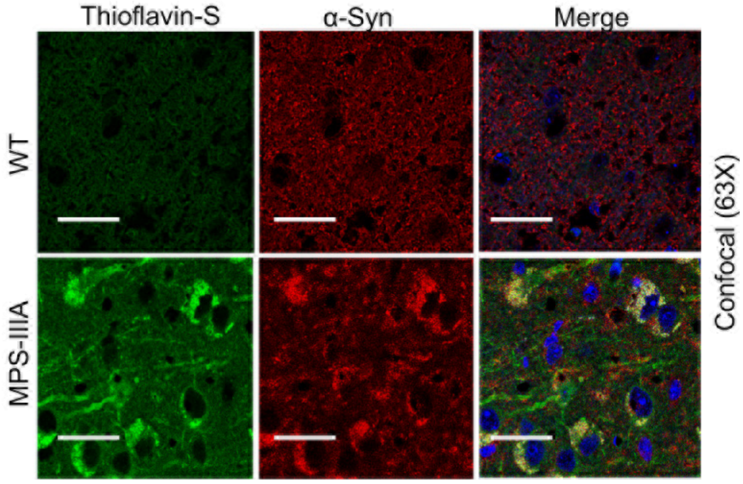

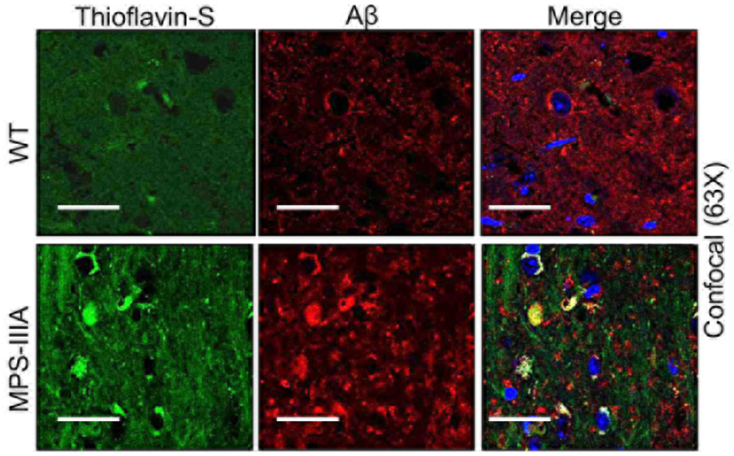

Supplementary Figure S4

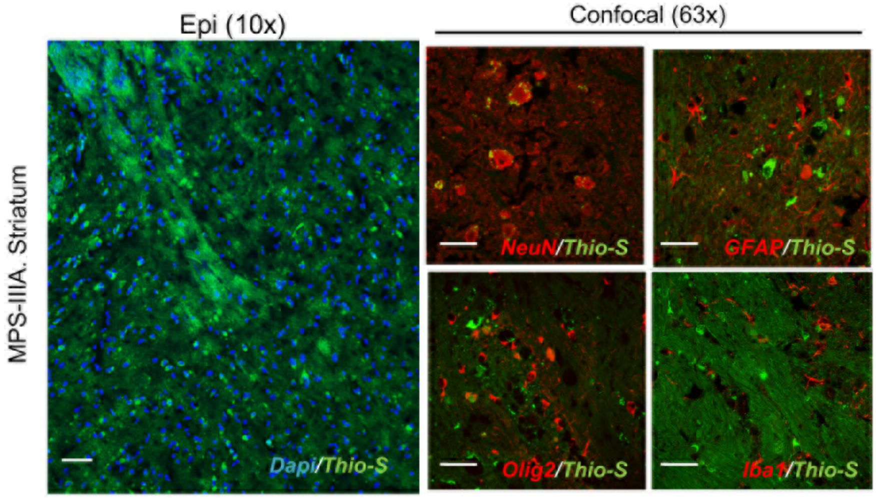

Supplementary Figure S5

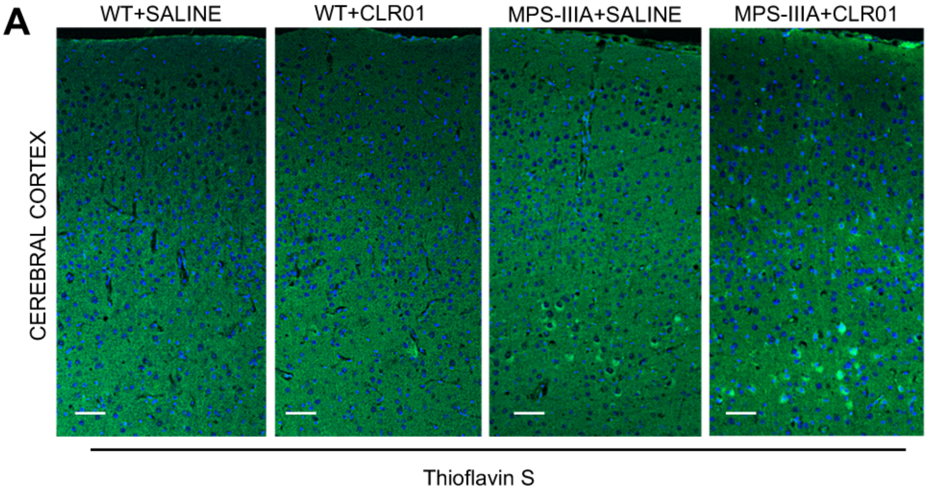

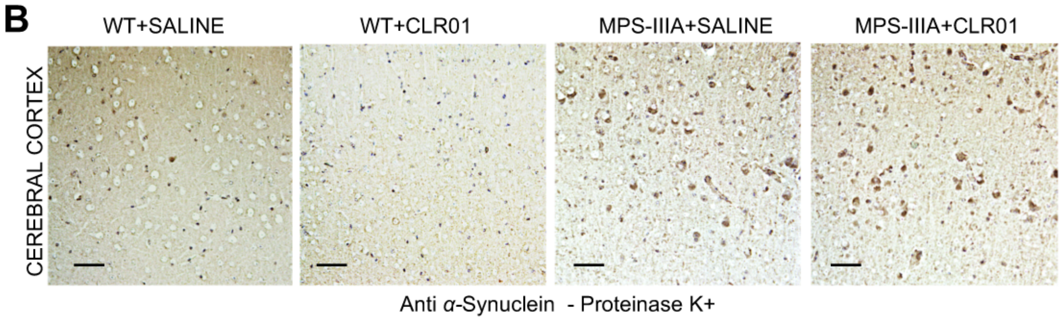

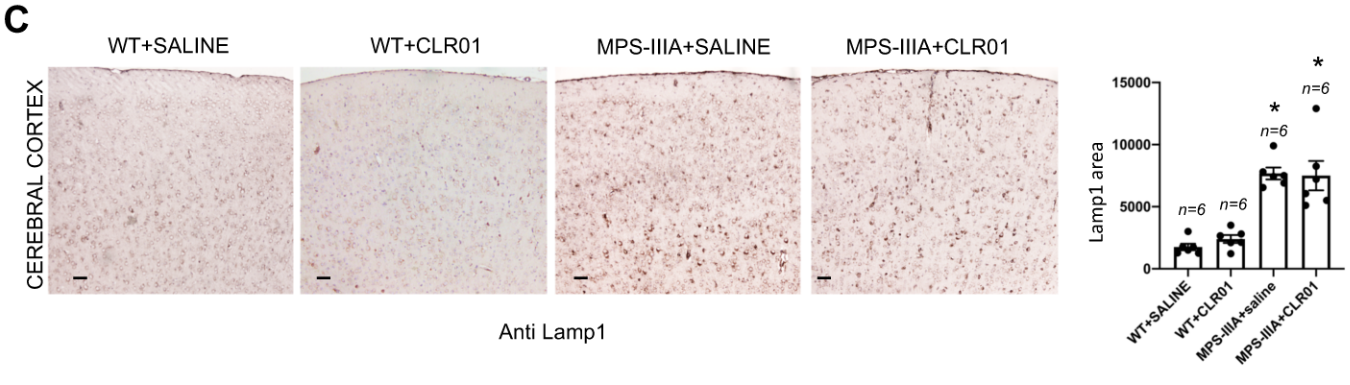

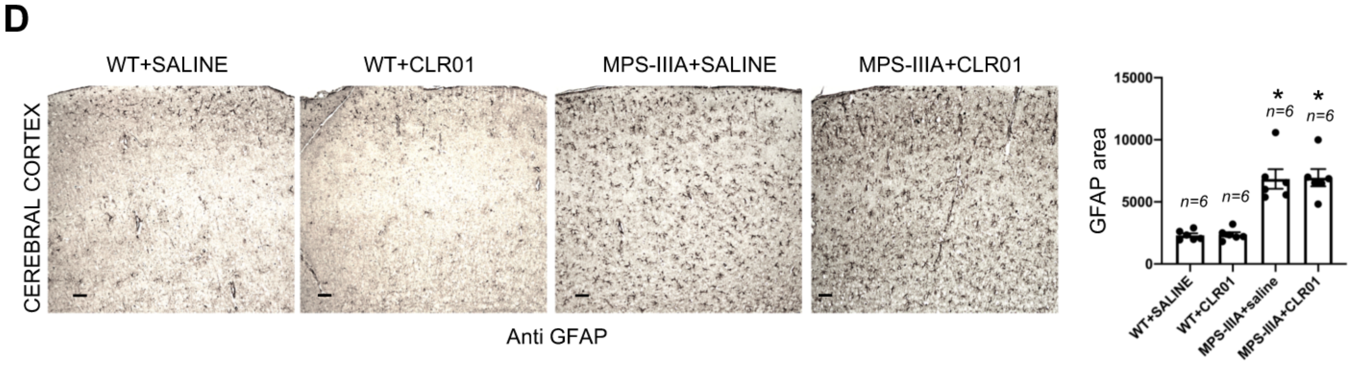

Supplementary figure S1. ***Congo Red staining in the brain of MPS-IIIA mice***

Congo Red staining was performed on cortical sagittal sections derived from WT and MPS-IIIA mice (9 months of age).

The scale bar represents 25 μm.

Supplementary figure S2. ***Further characterization of amyloidogenic protein aggregation in the brain of MPS-IIIA mice***

(A) Immunohistochemistry using an anti-α-synuclein antibody without or with proteinase-K treatment showing α-synuclein-positive signal in the hippocampus, midbrain, hypothalamus and cerebellum in WT and MPS-IIIA mice at 9 months of age.

(B) Immunohistochemistry experiments performed on WT and MPS-IIIA brain sections (9 months of age) with proteinase-K treatment using specific antibodies against PrP, tau or Aβ proteins showing the reactivity in the hippocampus, midbrain, hypothalamus and cerebellum.

(C) Immunohistochemistry experiments performed on WT or MPS-IIIA brain sections (9 months of age) with proteinase-K treatment using specific antibodies against TDP-43 or Huntingtin.

Scale bars represent 50μm (A, B, C) and 10 μm for the zoom images.

Supplementary figure S3: ***Confocal analysis of thioflavin S with specific amyloidogenic proteins***

Confocal-microscopy analysis on sagittal cortical brain sections from WT and MPS-IIIA mice (9 months of age) showing the colocalization between thioflavin-S (green) and either α-synuclein or Aβ (red).

Scale bars represent 10 μm.

Supplementary figure S4. ***Cell type localization of amyloid deposits in the striatum of MPS-IIIA mice***

Epi-fluorescence images (10x) show thioflavin S co-stained with DAPI in the striatum of MPS-IIIA mice at 9 months of age. Confocal images (63x) show thioflavin-S co-localization with neuronal (NeuN), astroglial (GFAP), oligodendrocyte (Olig2) or microglial (Iba1) markers in the same region.

Scale bar represent 25 µm in confocal-images and 50 µm in epi-fluorescence images.

Supplementary figure S5. ***CLR01 treatment at late stage of disease is not effective in ameliorating neuropathology in MPS-IIIA mice***

(A) CLR01 was injected daily at 1 mg/Kg subcutaneously to 7-months-old WT and MPS-IIIA mice over a period of 4 months. As a control, WT and MPS-IIIA mice were treated with saline. Thioflavin-S staining on sagittal sections derived from the indicated brain regions of the four experimental groups of mice (WT saline-injected, WT CLR01-injected, MPS-IIIA-saline injected and MPS-IIIA CLR01-injected) showed no significant reduction of amyloid deposition in the brain of CLR01 treated MPS-IIIA mice compared to controls.

(B) Immunohistochemistry with an anti-α-synuclein antibody with proteinase-K treatment on sagittal sections derived from the indicated brain regions of the four experimental groups of mice. Images of cortex show no significant reduction of proteinase-K resistant α-synuclein aggregates in MPS-IIIA mice upon CLR01 treatment.

(C) Immunohistochemistry with anti-Lamp1 antibody on sagittal sections derived from the indicated brain regions of mice belonging to the four experimental groups of mice. Images of cortex show no significant reduction of Lamp1 immunostained area in MPS-IIIA mice upon CLR01 treatment. Lamp1 immunoreactivity was quantified in 10-15 different fields taken from the cortical regions. Three mice for each experimental group were analysed.

*$p < 0.05$ *vs* WT + saline, one-way ANOVA with Bonferroni's multiple comparison test. Data are represented as means±SEM.

(D) Immunohistochemistry with anti-GFAP antibody on sagittal sections derived from the indicated brain regions of mice belonging to the four experimental groups of mice. Images of cortex show no significant reduction of both inflammatory markers in MPS-IIIA mice upon CLR01 treatment. GFAP immunoreactivity was quantified in 10-15 different fields taken from the cortical regions. Three mice for each experimental group were analysed.
*$p < 0.05$ *vs* WT + saline, one-way ANOVA with Bonferroni's multiple comparison test. Data are represented as means±SEM.
Scale bars in the figure represent 50 μm.

Supplementary table 1. ***Semi-quantitative analysis of amyloid deposition in the brain of MPS-IIIA mice***
Amyloid deposition was analyzed in the different brain regions of MPS-IIIA mouse brain (9 moths-old). Compact cytoplasmic, granular cytoplasmic, ring-shaped, axonal spheroids, tangle-like and filamentous inclusions were counted and their relative abundance was expressed by a score assignment: not detected (N.D.); few (+); numerous (++); abundant (+++).

Supplementary Table 1

Protein<br>Region	α-Syn	PrP	Aβ Peptide	tau	TDP-43	Htt
Cortex	Gc=++<br>Cc=++	Gc=++<br>S=+	ND	Tl=+<br>Cc=+	ND	ND
Striatum	Cc=++<br>S=+	Gc=+<br>S=+++	S=++	Cc=+<br>Rs=++<br>S=+	ND	ND
Hypothalamus	Gc=+<br>Cc=+<br>S=++	Gc=+<br>S=+++	S=+++	Cc=+<br>S=++	ND	ND
Hippocampus	Gc=++<br>Cc=++	ND	Gc=++<br>Cc=++	Gc=++	ND	ND
Midbrain	Cc=+<br>Gc=+<br>S=+	Gc=+<br>S=+	S=+	Cc=+<br>Fi=+	ND	ND
Cerebellum	Cc=+<br>Fi=+<br>S=+	Cc=+<br>S=+	S=+	Cc=+<br>Fi=+	ND	ND

Legend
Aggregation types:
Cc=Compact cytoplasmic; Gc=Granular cytoplasmic; Rs=Ring shaped (localized in the cell bodies).
S=Spheroids; Tl=Tangle like; Fi=Filamentous Inclusion (localized outside the cell bodies).
Quantification: +few, ++ numerous, +++ abundant, ND= Not Detected
